# Accuracy of plasma cell-free DNA PCR for non-invasive diagnosis of mucormycosis

**DOI:** 10.1128/jcm.00796-25

**Published:** 2025-07-09

**Authors:** Jordan Mah, Anthony Lieu, Veronica Nicholas, Angel Moreno, Kanagavel Murugesan, Indre Budvytiene, Niaz Banaei

**Affiliations:** 1 Department of Pathology, Stanford University School of Medicine 158566, Stanford, California, USA; 2 Clinical Microbiology Laboratory, Stanford Health Care, Stanford, California, USA; 3 Department of Medicine, Maisonneuve-Rosemont Hospital, Université de Montréal 5622, Montreal, Québec, Canada; 4 Research Center of Maisonneuve-Rosemont Hospital, Université de Montréal 5622, Montreal, Québec, Canada; 5 Division of Infectious Diseases, University of British Columbia 8166, Vancouver, British Columbia, Canada; 6 Division of Infectious Diseases and Geographic Medicine, Stanford University School of Medicine 196261 , Stanford, California, USA; 7 Division of Microbiology, University of British Columbia 8166, Vancouver, British Columbia, Canada; University of Utah, Salt Lake City, Utah, USA

**Keywords:** invasive Mucorales infection, immunocompromised hosts, cell-free DNA PCR, non-invasive diagnostic testing

## Abstract

Diagnosis of mucormycosis poses a substantial challenge due to the lack of a non-invasive biomarker and limitations of conventional diagnostics using invasive specimens. The aim of this study was to characterize the performance of Mucorales plasma cell-free DNA (cfDNA) PCR in immunosuppressed and non-immunosuppressed patients with pulmonary, disseminated, and localized mucormycosis. Patients with Mucorales plasma cfDNA test results were retrospectively categorized as proven or probable for sensitivity analysis and as no Mucorales infection for specificity analysis. In total, 85 positive and 212 negative Mucorales plasma cfDNA test results in unique patients were included in this study. Per the expert definitions, 47 patients had proven or probable mucormycosis, and 171 did not have invasive Mucorales infection. The Mucorales plasma cfDNA PCR had an overall sensitivity and specificity of 85.1% (40/47, 95% CI, 71.7–93.8) and 92.9% (158/170, 95% CI, 88.0–96.3), respectively. The sensitivity was 92.1% (35/38, 95% CI, 78.6–98.3) and 55.6% (5/9, 95% CI, 21.2–86.3) in patients with and without immunosuppression, respectively, and 100% (14/14, 95% CI, 76.8–100), 89.5% (17/19, 95% CI, 66.9–98.7), and 64.3% (9/14, 95% CI, 35.1–87.2) in patients with disseminated, pulmonary, and localized mucormycosis, respectively. Mucorales plasma cfDNA PCR is a sensitive and specific non-invasive testing modality for the diagnosis of mucormycosis in immunosuppressed patients and those with pulmonary and disseminated infection.

**IMPORTANCE:**

Mucormycosis is an invasive mold infection associated with high morbidity and mortality. Early diagnosis and effective antifungal treatment are critical for improving clinical outcomes. However, diagnosis of mucormycosis is often delayed due to the lack of a non-invasive biomarker and insensitivity of culture and non-specificity of histopathology performed on invasive specimens. Mucorales plasma cell-free DNA (cfDNA) PCR is a novel testing modality that allows non-invasive diagnosis of mucormycosis. In the current study, we evaluated the clinical performance of a pre-analytically optimized Mucorales plasma cfDNA PCR in immunosuppressed and non-immunosuppressed patients with pulmonary, disseminated, and localized infections. We show that Mucorales plasma cfDNA PCR is highly sensitive and specific in immunosuppressed patients with pulmonary and disseminated infections. Thus, the Mucorales plasma cfDNA PCR represents an accurate diagnostic tool for non-invasive diagnosis of mucormycosis, which may enable early treatment and improved outcomes in immunosuppressed patients with mucormycosis.

## INTRODUCTION

Mucormycosis is a life-threatening invasive fungal disease (IFD) caused by fungi belonging to the Mucorales order, with most infections being caused by a few genera, including *Rhizopus*, *Mucor*, and *Rhizomucor* (1). *Lichtheimia* is also a common Mucorales agent in some Asian and European countries (2, 3). Over the past decade, the global incidence of mucormycosis has increased due to a growing number of patients with uncontrolled diabetes, a rising number of patients receiving organ transplants and immunosuppressive therapies, and the emergence of COVID-19-associated mucormycosis (1, 4–6). Mucormycosis is associated with high morbidity and mortality in immunosuppressed patients, those with diabetic ketoacidosis, and patients with extensive burns (1, 7). Furthermore, delays in the initiation of appropriate antifungal therapy and surgical intervention have been linked to increased mortality, thus underscoring the critical importance of early and accurate diagnosis of mucormycosis (8). However, there is significant overlap in clinical presentation between mucormycosis and other types of IFD, such as invasive aspergillosis, thus underscoring the need for rapid and accurate diagnostics to initiate appropriate clinical management of mucormycosis (9).

Diagnosis of Mucorales infection poses substantial challenges due to the limitations of conventional diagnostics such as culture and histopathology, which require invasive sample collection and suffer from low sensitivity and specificity, respectively (10–12). Additionally, non-invasive fungal biomarkers, such as serum galactomannan and β-D-glucan, which can be helpful in the diagnosis of other causes of IFD, offer no utility in the diagnosis of mucormycosis (13). Among novel non-invasive testing modalities, cell-free DNA (cfDNA) PCR has been shown to be more accurate than conventional methods in diagnosing mucormycosis and prognosticating survival (14–17). Studies have demonstrated the ability of serum Mucorales PCR to diagnose mucormycosis earlier than conventional assays in patients with hematological malignancies and those in critical care settings for whom invasive procedures are often not possible (14, 15). However, Mucorales cfDNA PCR has only been evaluated in a few centers, and thus, more studies are needed to understand the performance characteristics of cfDNA PCR in different locales and different patient populations. We have previously described the pre-analytical optimization and clinical implementation of a mold plasma cfDNA PCR panel at our health system for routine testing of patients with suspected IFD (18). In the case of invasive aspergillosis, *Aspergillus* cfDNA PCR was shown to be significantly more sensitive than serum galactomannan and has been demonstrated to be highly concordant for IFD with results obtained from invasive sampling (19, 20).

The aim of this study was to evaluate the performance of optimized Mucorales plasma cfDNA PCR for the diagnosis of mucormycosis in a real-world setting where immunosuppressed and non-immunosuppressed patients with pulmonary, disseminated, and localized infections were tested by their providers.

## MATERIALS AND METHODS

### Study design

A retrospective cohort study was conducted at Stanford Health Care from September 2020 to May 2024 in adult and pediatric patients who underwent Mucorales plasma cfDNA testing for suspected IFD. All patients with positive cfDNA PCR results for Mucorales were included. Patients with negative PCR results were randomly selected at a ratio of 2:1 (negative to positive). In addition, patients who had a negative Mucorales plasma cfDNA PCR result but had a positive test result for Mucorales from a tissue biopsy, sterile body fluid, and/or bronchoalveolar lavage (BAL) fluid by nucleic acid amplification tests (PCR or targeted fungal sequencing) and/or fungal culture were also included. If patients had multiple negative plasma cfDNA PCR results, only their first result was included, and for those with multiple positive results, only their first positive result was included.

**Fig 1:**
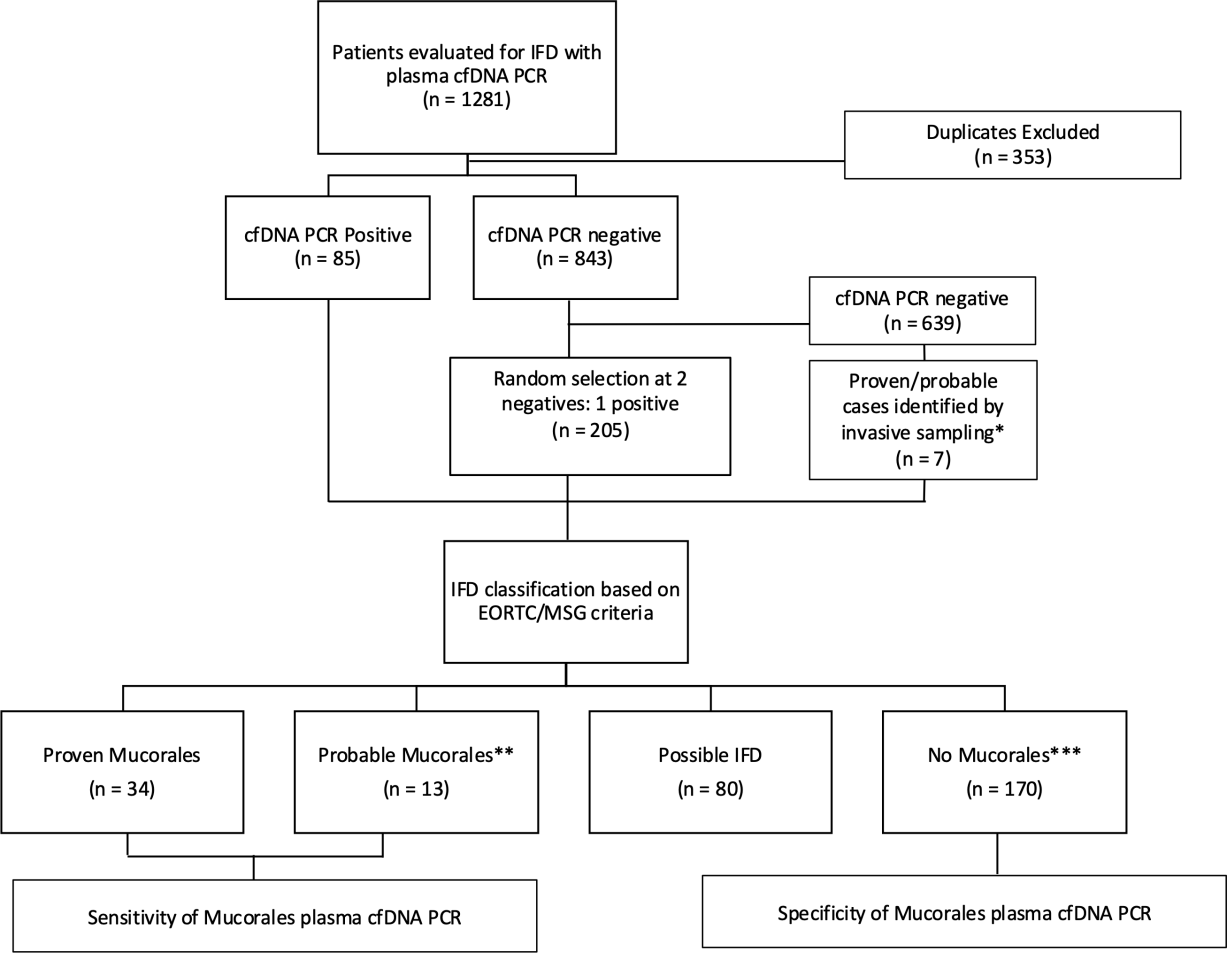
Study design and classification of cases in this study. Classification of cases was based on the EORTC/MSG definitions. *Positive by culture or molecular testing on tissue, sterile body fluid, and/or bronchoalveolar lavage fluid. **EORTC mycological criteria for probable Mucorales were extended to include two consecutive plasma cfDNA PCR. ***Included proven or probable invasive fungal disease caused by another mold.

Primary outcomes included clinical sensitivity and specificity of Mucorales cfDNA PCR. Proven and probable mucormycosis cases as defined by the European Organization for Research and Treatment of Cancer and the Mycoses Study Group Education and Research Consortium (EORTC/MSGERC) criteria were used as the true positive reference standard for sensitivity measurement, while no IFD or proven/probable IFD caused by another mold as defined by the EORTC/MSGERC was used as the true negative reference standard for specificity calculation (21). Similar to the EORTC/MSGERC definition for probable invasive aspergillosis, the definition of mycological criteria for probable mucormycosis was extended to include two consecutive positive plasma cfDNA PCR results, two consecutive positive BAL fluid PCR results, or one of each. Possible cases of IFD included appropriate host factors with clinical evidence of mold infection but without mycological evidence, which may have included single or non-consecutive positive plasma PCR results. No Mucorales IFD referred to the category of patients who did not meet the criteria for proven, probable, or possible IFD due to Mucorales. The secondary study endpoints included positive predictive value (PPV) and negative predictive value (NPV) at a prevalence of 5% and 20%, positive likelihood ratio (PLR) and negative likelihood ratio (NLR), and diagnostic odds ratios (DORs). A diagram describing the study design is shown in Fig. 1.

### Clinical data

An electronic report was used to identify patients who had undergone fungal testing. The electronic medical records were reviewed to collect patient demographics and data on comorbidities, radiologic findings, microbiological results, antifungal treatments, and clinical outcomes. The patients were categorized into proven, probable, possible, and no mucormycosis based on the consensus definition by the EORTC/MSGERC. Consecutive positives were defined as two or more positive PCR results within 7 days. Clinical presentations of mucormycosis were categorized as pulmonary, localized (cutaneous and rhino-orbital), and disseminated (two or more non-contiguous sites). Suspected pulmonary infection was defined as abnormal chest diagnostic imaging.

### Mucorales PCR

The Mucorales plasma cfDNA PCR is a laboratory-developed test performed daily in the Stanford Health Care clinical microbiology laboratory using optimized pre-analytical and analytical parameters previously described (18, 22). It is a singleplex real-time PCR assay that detects *Rhizopus* spp., *Rhizomucor* spp., and *Mucor* spp. targeting the 18S rRNA, using primers (NS92F CACCGCCCGTCGCTAC; Muc-R1-A CCTAGTTTGCCATAGTTCTCTGCAG; and RmucR1 GTAGTTTGCCATAGTTCGGCTA), probes (RmucP1 CAL Fluor Red 610-AATGGCTATAGTGAGCATATGGGAGGCT-BHQ-2; MucP1 CAL Fluor Red 610-CGATTGAATGGTTATAGTGAGCATATGGG-BHQ-2), and PCR conditions previously described (18). It is part of a mold PCR panel, which includes singleplex and multiplex PCR reactions detecting the most common mold pathogens (*Aspergillus* spp.*, Fusarium* spp.*, Scedosporium* spp., *Lomentospora prolificans*, and Mucorales agents). The human beta-globin gene was used as an internal control for DNA extraction and PCR inhibition. A Mucorales PCR cycle threshold <40 was considered positive. Samples with cycle thresholds 40–45 were repeated for confirmation and reported as positive only if reproducible. A cycle threshold was reported in addition to the qualitative result.

### Data analysis

Continuous data were summarized as means with standard deviation (SD) and compared using *t* test. Categorical data were compared using the Fisher’s exact test. A *P*-value of <0.05 was considered statistically significant. SPSS version 28 (IBM, Chicago, IL, USA) was used for analysis.

## RESULTS

### Patient characteristics

Out of a total of 1,281 Mucorales plasma cfDNA PCR tests performed in 928 unique patients during the study period, 85 positive and 212 negative tests in unique patients were included in this study. The negatives included 205 randomly selected results and 7 results from proven or probable cases based on positive results with conventional diagnostics performed on tissue, sterile body fluid, and/or BAL samples (Fig. 1). The study cohort consisted of 54.2% male with a mean age of 52.1 years old (SD ± 23.6). Two hundred sixty-two (88.2%) patients were immunosuppressed due to hematologic malignancy (HM, 28.3%), hematopoietic stem cell transplant (HSCT, 26.3%), solid organ transplantation (SOT, 16.8%), solid organ malignancy (7.7%), and other causes (9.1%). Among the 35 non-immunosuppressed patients, 11 (31.4%) had diabetes. In terms of clinical presentation, suspected pulmonary infection was the most common (231, 77.8%), followed by localized infection (31, 10.4%), disseminated infection (28, 9.4%), and unknown (7, 2.4%). Invasive specimen collection for conventional testing was performed in 45.8% of patients (Table 1). Mold prophylaxis with activity against Mucorales was administered in 42.1% of patients.

**TABLE 1:** Patient characteristics and demographics^*c*^

	Overall (*n* = 297)	Mucorales cfDNA PCR positive (*n* = 85)	Mucorales cfDNA PCR negative (*n* = 212)
Mean age ± SD	52.1 ± 23.6	49.6 ± 25.4	51.2 ± 21.4
Sex, number (%)			
Male	160 (53.9)	40 (47.1)	120 (56.6)
Immunosuppression, number (%)	262 (88.2)	74 (87.1)	188 (88.7)
Type of immunosuppression, number (%)			
Hematological malignancy	84 (28.3)	32 (37.6)	52 (24.5)
Hematopoietic stem cell transplant	78 (26.3)	21 (24.7)	57 (26.9)
Solid organ transplantation	50 (16.8)	9 (10.6)	41 (19.3)
Solid organ malignancy	23 (7.7)	2 (2.4)	21 (9.9)
Other^*a*^	27 (9.1)	10 (11.7)	17 (8.0)
None	35 (11.8)	11 (12.9)	24 (11.3)
Clinical presentation, number (%)			
Pulmonary	231 (77.8)	55 (64.7)	176 (83.0)
Disseminated	28 (9.4)	16 (18.8)	12 (5.7)
Localized (sinus/skin)	31 (10.4)	11 (12.9)	20 (9.4)
Unknown	7 (2.4)	3 (3.5)	4 (1.9)
Mold prophylaxis, number (%)	124 (41.8)	48 (56.5)	76 (35.8)
Invasive procedure performed, number (%)	135 (45.5)	48 (56.5)	87 (41.0)
30-day all-cause mortality^*b*^ number (%)	81 (27.3)	41 (48.2)	40 (18.9)
Diagnosis of invasive Mucorales based on EORTC/MSGERC Criteria, number (%)			
Proven Mucorales	34 (11.4)	27 (31.8)	7 (3.3)
Probable Mucorales	13 (4.3)	12 (14.1)	1 (0.5)
Possible IFD	80 (26.9)	34 (40.0)	46 (21.7)
No Mucorales	170 (57.2)	12 (14.1)	158 (74.5)

^*a*^ Other refers to HIV and immunosuppression, which did not include HM/HSCT/SOT.

^*b*^ From the time of mold plasma cfDNA PCR.

^*c*^ SD – standard deviation; cfDNA PCR – cell-free DNA polymerase chain reaction .

According to the EORTC/MSGERC definitions for mucormycosis, 34 (11.4%) patients had proven, 13 (4.3%) had probable, 80 (26.9%) had possible, and 170 (57.2%) had no invasive mold disease (IMD) due to Mucorales. Among the cases categorized as probable, five had positive fungal respiratory cultures for Mucorales. Using the extended criteria, one case had both a positive plasma cfDNA PCR and a positive BAL PCR for Mucorales, while seven cases met mycological criteria only based on consecutive positive plasma cfDNA PCR results; all seven were treated for invasive mucormycosis. Of the 170 cases with no mucormycosis, 54 had proven/probable IFD due to another mold, while 116 had no IMD. There were four mixed infections in the cohort, including two proven mucormycosis and aspergillosis, one probable mucormycosis and proven aspergillosis, and one probable mucormycosis and aspergillosis. The overall 30 day all-cause mortality of the cohort was 27.3%. In proven and probable mucormycosis cases, the 30 day all-cause mortality rate was 41.3%.

### Accuracy of Mucorales plasma cfDNA PCR

The Mucorales plasma cfDNA PCR had an overall sensitivity of 85.1% (40/47, 95% CI, 71.7–93.8) in proven or probable cases. When the analysis was stratified by patients’ immune status and clinical presentation, the sensitivity was 92.1% (35/38, 95% CI, 78.6–98.3) and 55.6% (5/9, 95% CI, 21.2–86.3) in patients with and without immunosuppression, respectively, and 100% (14/14, 95% CI, 76.8–100) in patients with disseminated IFD, 89.5% (17/19, 95% CI, 66.9–98.7) in patients with pulmonary disease, and 64.3% (9/14, 95% CI, 35.1–87.2) in patients with localized infection (Table 2). When localized infection was stratified by patient’s immune status, the sensitivity was 83.3% (5/6, 95% CI, 35.9–99.6) in immunosuppressed patients and 50.0% (4/8, 95% CI, 15.7–84.3) in patients without immunosuppression. The overall sensitivity of Mucorales plasma cfDNA PCR in patients on mold prophylaxis was 92.9% (26/28, 95% CI, 76.5–99.1) (Table 2).

**TABLE 2:** Sensitivity and specificity of Mucorales plasma cfDNA PCR^*a*^

		Patient population				Clinical presentation		
	Overall	IS^*b*^	HM/HSCT	SOT/other^*c*^	Not IS	Pulmonary	Disseminated	Localized
Mucorales plasma cfDNA PCR sensitivity (*n*/*N*, 95% confidence interval)								
Definition of true positive								
Proven IFD	82.4 (28/34, 95% CI, 65.5–93.2)	92.0 (23/25, 95% CI, 74.0–99.0)	88.2 (15/17, 95% CI, 63.6–98.5)	100 (8/8, 95% CI, 63.1–100)	55.6 (5/9, 95% CI, 21.2–86.3)	88.9 (8/9, 95% CI, 51.8–99.7)	100 (12/12, 95% CI, 73.5–100)	61.5 (8/13, 95% CI, 31.6–86.1)
Probable IFD	92.3 (12/13, 95% CI, 64.0–99.8)	92.3 (12/13, 95% CI, 64.0–99.8)	100 (9/9, 95% CI, 66.4–100)	75 (3/4, 95% CI, 19.4–99.4)	NA	90 (9/10, 95% CI, 55.5–99.8)	100 (2/2, 95% CI, 15.8–100)	100 (1/1, 95% CI, 25–100)
Proven or probable IFD	85.1 (40/47, 95% CI, 71.7–93.8)	92.1 (35/38, 95% CI, 78.6–98.3)	92.3 (24/26, 95% CI, 74.9–99.1)	91.7 (11/12, 95% CI, 61.5–99.8)	55.6 (5/9, 95% CI, 21.2–86.3)	89.5 (17/19, 95% CI, 66.9–98.7)	100 (14/14, 95% CI, 76.8–100)	64.3 (9/14, 95% CI, 35.1–87.2)
Proven or probable IFD on mold prophylaxis	92.9 (26/28, 95% CI, 76.5–99.1)	92.9 (26/28, 95% CI, 76.5–99.1)	95.5 (21/22, 95% CI, 77.2–99.9)	83.3 (5/6, 95% CI, 35.9–99.6)	NA	87.5 (14/16, 95% CI, 61.7–98.5)	100 (10/10, 95% CI, 69.2–100)	100 (2/2, 95% CI, 15.7–100)
Mucorales plasma cfDNA PCR specificity (*n*/*N*, 95% confidence interval)								
Definition of true negative								
No Mucorales IFD	92.9 (158/170, 95% CI, 88.0–96.3)	93.9 (139/148, 95% CI, 88.8–97.2)	92.0 (80/87, 95% CI, 84.1–96.7)	93.6 (44/47, 95% CI, 82.5–98.7)	86.4 (19/22, 95% CI, 82.5–98.7)	93.4 (128/137, 95% CI, 87.9–97.0)	100 (12/12, 95% CI, 73.5–100)	93.3 (14/15, 95% CI, 68.1–99.8)
No Mucorales IFD on mold prophylaxis	93.0 (53/57, 95% CI, 83.0–98.1)	91.2 (52/57, 95% CI, 80.7–97.1)	88.9 (32/36, 95% CI, 73.9–96.9)	95.2 (20/21, 95% CI, 76.2–99.9)	NA	91.3 (42/46, 95% CI, 79.2–97.6)	100 (7/7, 95% CI, 59.0–100)	100 (4/4, 95% CI, 39.8–100)

^*a*^ CI, confidence intervals; SOT, solid organ transplantation; IS, immunosuppression; and NA, not applicable.

^*b*^ IS refers to immunosuppressed patient populations including HM, HSCT, SOT, and others.

^*c*^ Other refers to immunosuppressed groups, which included HIV/AIDS, auto-immune, and rheumatological disorders.

The overall specificity of Mucorales plasma cfDNA PCR in patients without mucormycosis was 92.9% (158/170, 95% CI, 88.0–96.3). The specificity was similar in patients on mold prophylaxis and across different immune status and clinical presentations (Table 2).

At a prevalence of 5%, the Mucorales cfDNA overall PPV was 38.8% (26.6–52.6) and NPV was 99.2% (98.4–99.6); at 20% prevalence, the overall PPV was 75.1% (63.3–84.1) and NPV was 96.2 (92.6–98.0) (Table 3). The overall PLR and NLR for proven or probable Mucorales were 12.1 and 0.2, respectively, with a DOR of 75.4. Sub-analysis for different groups is shown in Table 3.

**TABLE 3:** Performance characteristics of Mucorales plasma cfDNA PCR for the diagnosis of mucormycosis^*a*^

	Mucorales plasma cfDNA PCR				
	Patient population				
	Overall	IS^*b*^	HM/HSCT	SOT/other^*c*^	No IS
PPV^*d*^					
Prevalence 5%	38.8 (26.6–52.6)	44.4 (29.6–60.2)	37.7 (22.7–55.3)	43.1 (20.0–69.6)	17.7 (6.1–41.7)
Prevalence 20%	75.1 (63.3–84.1)	79.1 (66.6–87.8)	74.2 (58.3–85.5)	99.5 (97.0–99.9)	97.4 (94.6–98.7)
NPV^*d*^					
Prevalence 5%	99.2 (98.4–99.6)	99.6 (98.7–99.9)	99.6 (98.4–99.9)	78.2 (54.2–91.6)	50.5 (23.4–77.2)
Prevalence 20%	96.2 (92.6–98.0)	97.9 (94.1–99.3)	98.0 (92.7–99.5)	97.8 (87.3–99.7)	88.6 (78.6–94.3)
Positive LR (sensitivity/1 – specificity)	12.1	15.3	11.5	14.3	4.0
Negative LR (1 – sensitivity/specificity)	0.2	0.1	0.1	0.1	0.5
DOR (positive LR / negative LR)	75.4	189.4	143.4	159.6	8.0

^*a*^ SOT, solid organ transplantation and LR, likelihood ratio.

^*b*^ Probable IFI, which met criteria by two consecutive positive PCR results as the mycological criteria, was excluded from calculation for Mucorales plasma cfDNA PCR.

^*c*^ Other refers to immunosuppression, which did not include HM/HSCT/SOT.

^*d*^ Reported as percentage with 95% confidence intervals.

### Investigation of false cfDNA PCR results

In total, seven patients with proven or probable mucormycosis had false-negative Mucorales plasma cfDNA PCR results. The clinical and laboratory findings of these patients are presented in Table 4. They consisted of two *Rhizopus* spp. and one *Mucor* spp. identified by culture and fungal sequencing, and four Mucorales agents identified by the Mucorales PCR. The latter four cases had positive histopathology findings consistent with Mucorales infection. Five of these patients were not considered immunosuppressed according to EORTC/MSG criteria but had diabetes (57.1%) or a recent COVID-19 infection (57.1%) as their underlying comorbidity. Five patients had localized sinusitis or cutaneous mucormycosis, and two had pulmonary mucormycosis.

**TABLE 4:** Summary of clinical and microbiological data for patients with false-negative Mucorales plasma cfDNA PCR results^*a*^

Patient number	Underlying comorbidity	Infection site, EORTC classification	Histopathology results^*b*^	Fungal etiology	Microbiology results	Treatment	Mortality 30 days/6 months
1	Lung transplant	Lung, probable	Negative	*Rhizopus* spp.	Positive BAL culture with 10 colonies of *Rhizopus* spp., BAL GM 1.55	Isavuconazole	N/N
2	Diabetes, COVID-19	Sinus, proven	Positive	*Rhizopus, Mucor,* or *Rhizomucor* spp.	Positive Mucorales PCR on tissue, negative tissue culture	L-amphotericin and debridement	N/Y
3	Diabetes	Sinus, proven	Positive	*Rhizopus, Mucor,* or *Rhizomucor* spp.	Positive Mucorales PCR on paraffin-embedded tissue, negative tissue culture	L-amphotericin, micafungin, and debridement	N/N
4	Diabetes, COVID-19	Lung, proven	Positive	*Rhizopus, Mucor,* or *Rhizomucor* spp.	Positive Mucorales PCR on paraffin-embedded tissue, negative tissue culture	L-amphotericin and lobectomy	N/N
5	Diabetes, COVID-19	Sinus, proven	Positive	*Rhizopus, Mucor,* or *Rhizomucor* spp.	Positive Mucorales PCR on paraffin-embedded tissue	L-amphotericin and debridement	N/N
6	Esophageal malignancy, COVID-19	Sino-orbital, proven	Positive	*Rhizopus* spp.	Positive fungal sequencing on paraffin-embedded tissue for *Rhizopus* spp.	L-amphotericin and debridement	N/N
7	AML	Finger, proven	Positive	*Mucor* spp.	Positive tissue culture with one colony of Mucor spp.	L-amphotericin and amputation	N/N

^*a*^ IA, invasive aspergillosis; ARDS, acute respiratory distress syndrome; AML, acute myeloid leukemia; OD, optical density; coronavirus disease, COVID-19.

^*b*^ Histopathology results refer to the presence of fungal hyphal elements.

Among 12 patients with false-positive Mucorales cfDNA PCR results, 5 did not have a clinical or radiographic feature to support a diagnosis of IMD, and 10 had an alternative diagnosis. All 12 had a single positive Mucorales cfDNA PCR test, with 10 having a cycle threshold value of ≥38. Clinical and laboratory findings of patients with false-positive Mucorales cfDNA PCR are presented in Table S1.

### Significance of single positive PCR results

There were 34 possible cases with a single positive plasma cfDNA PCR result, and the 30-day all-cause mortality rate in this group was 55.9%, which was not statistically different from proven and probable cases (*P* = 0.12), but it was significantly higher compared to cases that tested negative with Mucorales cfDNA PCR (18.9%; *P* < 0.01).

## DISCUSSION

Mucormycosis in immunocompromised hosts is associated with high morbidity and mortality, thus making timely diagnosis crucial for the initiation of effective antifungal therapy and surgical management with resection (7, 9). Conventional diagnostics such as fungal culture, and histopathology are slow and require invasive sampling of tissue, which is impractical in patients who cannot tolerate invasive procedures. In this study, a Mucorales plasma cfDNA PCR that has been pre-analytically optimized was shown to be 92.1% sensitive in immunosuppressed patients, and 100% and 89.5% sensitive in disseminated and pulmonary infections, respectively. However, it was less sensitive in non-immunosuppressed patients, particularly in those with localized infection. Although *Lichtheimia* spp., which are not targeted in the Mucorales PCR assay investigated in this study, have been shown to cause cutaneous articular infections in non-immunosuppressed patients, the false-negative cases in this study were all attributed to Mucorales genera that were targeted on the panel (23). The difference in sensitivities in immunosuppressed versus non-immunosuppressed and in patients with different comorbidities likely reflects the immunopathogenesis of mucormycosis, leading to varying levels of Mucorales cfDNA in the blood (9, 24). Although Mucorales cfDNA PCR has demonstrated success in some non-immunosuppressed hosts such as burn patients, many of these patients had burns covering over 60% of their body surface area, which may explain the high fungal burden and detectable plasma cfDNA in these patients (25). Although the sensitivity of Mucorales plasma cfDNA PCR was low in localized rhino-orbital mucormycosis in diabetic patients, these cases present fewer diagnostic challenges compared to pulmonary mucormycosis in immunocompromised patients (1, 13). These patients do not typically have contraindications for surgical biopsy, and obtaining a biopsy from the sinuses poses fewer challenges compared with pulmonary or gastrointestinal sites (12, 26). Furthermore, pulmonary mucormycosis has many radiographic and clinical features that overlap with those seen in bacterial, mycobacterial, and other invasive mold diseases, which makes identification of the mold essential for choosing appropriate antifungal therapy. In contrast, rhino-orbital fungal infection in diabetics is most commonly caused by Mucorales agents. In these cases, surgical resection is also essential for effective management. Consequently, despite the lower sensitivity of Mucorales plasma cfDNA PCR in diabetics with rhino-orbital mucormycosis, the use of non-invasive diagnostics in this population is not as critical in clinical practice compared with patients with pulmonary infection.

The overall sensitivity of Mucorales plasma cfDNA PCR in the current study is consistent with sensitivities reported in prior studies using serum or plasma, ranging from 73.3% to 100% (14, 27–29). However, these studies have been limited by small sample sizes, which hindered sub-analyses in patients with different comorbidities and clinical presentations (14, 27–29). A key strength of the current study was the investigation of the sensitivity of the Mucorales plasma cfDNA PCR in immunosuppressed and non-immunosuppressed patients, and patients with rhino-orbital and skin mucormycosis. While the sensitivity was high (92.1%) in immunosuppressed hosts, it was lower in non-immunosuppressed patients (55.6%), especially in those with localized sinus or cutaneous infection (64.3%). However, when we further stratified the patients with localized infection by immune status, the sensitivity of plasma cfDNA PCR remained moderately high (83.3%) in immunosuppressed patients. This finding indicates that the host immune status is the major determinant of the sensitivity of the Mucorales plasma cfDNA PCR even when infection is localized. Another novel aspect of this study was investigating the sensitivity of Mucorales plasma cfDNA PCR in patients on mold prophylaxis. Being on active mold prophylaxis could decrease the sensitivity of cfDNA PCR, as there may be a lower fungal burden and therefore less detectable cfDNA present in the plasma. However, we found that Mucorales plasma cfDNA PCR remained highly sensitive (92.9%) despite being on “Mucorales active” mold prophylaxis. Furthermore, we showed that Mucorales plasma cfDNA PCR had a high specificity (92.9%), which is consistent with prior studies reporting specificities ranging from 89.8% to 100% (14, 27–29). Another novel aspect of the current study was that specificity remained high (91.2%) in patients on mold prophylaxis. This is relevant because prior studies have reported lower specificity for *Aspergillus* cfDNA PCR in patients on mold prophylaxis, but we have not observed lower specificities for either *Aspergillus* or Mucorales in patients on mold prophylaxis (19, 30–32).

This study was strengthened by using a large cohort of proven and probable mucormycosis cases to investigate the accuracy of Mucorales plasma cfDNA PCR. However, there are several limitations that may impact its findings. First, the retrospective single-center design of this study may limit the generalizability of its findings to other health systems with different patient populations and testing practices. Second, the Mucorales genera targeted in our assay were selected based on our local and national epidemiology (2, 3, 33, 34). Predominant Mucorales agents at our institution are *Rhizopus* spp., *Rhizomucor* spp., and *Mucor* spp. Therefore, our reported sensitivities of Mucorales cfDNA PCR may not apply to settings in geographic areas where other Mucorales agents, such as *Lichtheimia* spp., are more prevalent. Third, we may have overestimated the sensitivity of Mucorales plasma cfDNA PCR due to the inclusion of consecutive Mucorales cfDNA PCR-positive cases as mycological evidence for probable cases. However, only seven cases met the probable definition based on consecutive positive cfDNA PCR, and all were treated for Mucorales infection, thus indicating that providers considered the PCR results clinically relevant. Furthermore, some groups have expressed that the cumulative experience with Mucorales PCR and the low interlaboratory variability demonstrated by recent studies on Mucorales PCR should be used to accelerate Mucorales PCR positivity as a criterion of the EORTC/MSGERC definition for probable cases (14). Finally, we may have also underestimated the sensitivity of Mucorales plasma cfDNA PCR by including all cases from patients with negative Mucorales plasma cfDNA PCR results but positive Mucorales molecular and culture results in tissue, sterile body fluid, and/or bronchoalveolar lavage fluid. A prospective design might have yielded a higher Mucorales plasma cfDNA PCR sensitivity.

In conclusion, Mucorales plasma cfDNA PCR is a promising non-invasive diagnostic tool for the accurate diagnosis of mucormycosis in immunocompromised hosts. It provides timely diagnosis, which is needed to improve outcomes. Further studies are needed to confirm its accuracy across diverse populations and clinical presentations.

## SUPPLEMENTAL MATERIAL

Table S1 jcm.00796-25-s0001.docx Table shows clinical and laboratory data for patients with false-positive Mucorales plasma cfDNA PCR results.

ASM does not own the copyrights to Supplemental Material that may be linked to, or accessed through, an article. The authors have granted ASM a non-exclusive, world-wide license to publish the Supplemental Material files. Please contact the corresponding author directly for reuse.

## References

[1] Petrikkos G, Skiada A, Lortholary O, Roilides E, Walsh TJ, Kontoyiannis DP. 2012. Epidemiology and clinical manifestations of mucormycosis. Clin Infect Dis 54 Suppl 1:S23–S34. doi:10.1093/cid/cir866 22247442

[2] Lanternier F, Dannaoui E, Morizot G, Elie C, Garcia-Hermoso D, Huerre M, Bitar D, Dromer F, Lortholary O, French Mycosis Study Group. 2012. A global analysis of mucormycosis in France: the RetroZygo Study (2005-2007). Clin Infect Dis 54 Suppl 1:S35–S43. doi:10.1093/cid/cir880 22247443

[3] Prakash H, Chakrabarti A. 2019. Global epidemiology of mucormycosis. J Fungi (Basel) 5:26. doi:10.3390/jof5010026 30901907 PMC6462913

[4] Douglas AP, Chen S-A, Slavin MA. 2016. Emerging infections caused by non-Aspergillus filamentous fungi. Clin Microbiol Infect 22:670–680. doi:10.1016/j.cmi.2016.01.011 26812445

[5] Bitar D, Van Cauteren D, Lanternier F, Dannaoui E, Che D, Dromer F, Desenclos JC, Lortholary O. 2009. Increasing incidence of zygomycosis (mucormycosis), France, 1997-2006. Emerg Infect Dis 15:1395–1401. doi:10.3201/eid1509.090334 19788806 PMC2819884

[6] Hoenigl M, Seidel D, Carvalho A, Rudramurthy SM, Arastehfar A, Gangneux J-P, Nasir N, Bonifaz A, Araiza J, Klimko N, Serris A, Lagrou K, Meis JF, Cornely OA, Perfect JR, White PL, Chakrabarti A, ECMM and ISHAM collaborators. 2022. The emergence of COVID-19 associated mucormycosis: a review of cases from 18 countries. Lancet Microbe 3:e543–e552. doi:10.1016/S2666-5247(21)00237-8 35098179 PMC8789240

[7] Roden MM, Zaoutis TE, Buchanan WL, Knudsen TA, Sarkisova TA, Schaufele RL, Sein M, Sein T, Chiou CC, Chu JH, Kontoyiannis DP, Walsh TJ. 2005. Epidemiology and outcome of zygomycosis: a review of 929 reported cases. Clin Infect Dis 41:634–653. doi:10.1086/432579 16080086

[8] Chamilos G, Lewis RE, Kontoyiannis DP. 2008. Delaying amphotericin B-based frontline therapy significantly increases mortality among patients with hematologic malignancy who have zygomycosis. Clin Infect Dis 47:503–509. doi:10.1086/590004 18611163

[9] Kontoyiannis DP, Lewis RE. 2006. Invasive zygomycosis: update on pathogenesis, clinical manifestations, and management. Infect Dis Clin North Am 20:581–607, doi:10.1016/j.idc.2006.06.003 16984870

[10] Lackner N, Posch W, Lass-Flörl C. 2021. Microbiological and molecular diagnosis of mucormycosis: from old to new. Microorganisms 9:1518. doi:10.3390/microorganisms9071518 34361953 PMC8304313

[11] Cornely O A, Arikan-Akdagli S, Dannaoui E, Groll AH, Lagrou K, Chakrabarti A, Lanternier F, Pagano L, Skiada A, Akova M, et al.. 2014. ESCMID and ECMM joint clinical guidelines for the diagnosis and management of mucormycosis 2013. Clin Microbiol Infect 20 Suppl 3:5–26. doi:10.1111/1469-0691.12371 24479848

[12] Cornely OA, Alastruey-Izquierdo A, Arenz D, Chen SCA, Dannaoui E, Hochhegger B, Hoenigl M, Jensen HE, Lagrou K, Lewis RE, et al.. 2019. Global guideline for the diagnosis and management of mucormycosis: an initiative of the European Confederation of Medical Mycology in cooperation with the Mycoses Study Group Education and Research Consortium. Lancet Infect Dis 19:e405–e421. doi:10.1016/S1473-3099(19)30312-3 31699664 PMC8559573

[13] Kontoyiannis DP, Lewis RE. 2011. How I treat mucormycosis. Blood 118:1216–1224. doi:10.1182/blood-2011-03-316430 21622653 PMC3292433

[14] Millon L, Caillot D, Berceanu A, Bretagne S, Lanternier F, Morio F, Letscher-Bru V, Dalle F, Denis B, Alanio A, Boutoille D, Bougnoux ME, Botterel F, Chouaki T, Charbonnier A, Ader F, Dupont D, Bellanger AP, Rocchi S, Scherer E, Gbaguidi-Haore H, Herbrecht R. 2022. Evaluation of serum mucorales polymerase chain reaction (PCR) for the diagnosis of mucormycoses: the MODIMUCOR prospective trial. Clin Infect Dis 75:777–785. doi:10.1093/cid/ciab1066 34986227

[15] Millon L, Herbrecht R, Grenouillet F, Morio F, Alanio A, Letscher-Bru V, Cassaing S, Chouaki T, Kauffmann-Lacroix C, Poirier P, Toubas D, Augereau O, Rocchi S, Garcia-Hermoso D, Bretagne S, French Mycosis Study Group. 2016. Early diagnosis and monitoring of mucormycosis by detection of circulating DNA in serum: retrospective analysis of 44 cases collected through the French Surveillance Network of Invasive Fungal Infections (RESSIF). Clin Microbiol Infect 22:810. doi:10.1016/j.cmi.2015.12.006 26706615

[16] Millon L, Larosa F, Lepiller Q, Legrand F, Rocchi S, Daguindau E, Scherer E, Bellanger A-P, Leroy J, Grenouillet F. 2013. Quantitative polymerase chain reaction detection of circulating DNA in serum for early diagnosis of mucormycosis in immunocompromised patients. Clin Infect Dis 56:e95–101. doi:10.1093/cid/cit094 23420816

[17] Moreno A, Mah J, Budvytiene I, Ho DY, Schwenk HT, Banaei N. 2024. Dynamics and prognostic value of plasma cell-free DNA PCR in patients with invasive aspergillosis and mucormycosis. J Clin Microbiol 62:e0039424. doi:10.1128/jcm.00394-24 38602412 PMC11237630

[18] Senchyna F, Hogan CA, Murugesan K, Moreno A, Ho DY, Subramanian A, Schwenk HT, Budvytiene I, Costa HA, Gombar S, Banaei N. 2021. Clinical accuracy and impact of plasma cell-free DNA fungal polymerase chain reaction panel for noninvasive diagnosis of fungal infection. Clin Infect Dis 73:1677–1684. doi:10.1093/cid/ciab158 33606010

[19] Mah J, Nicholas V, Tayyar R, Moreno A, Murugesan K, Budvytiene I, Banaei N. 2023. Superior accuracy of Aspergillus plasma cell-free DNA polymerase chain reaction over serum galactomannan for the diagnosis of invasive aspergillosis. Clin Infect Dis 77:1282–1290. doi:10.1093/cid/ciad420 37450614

[20] Lieu A, Zimmet AN, Pozdol J, Kushner LE, Ho D, Banaei N. 2025. Concordance of non-invasive plasma cell-free DNA with invasive diagnostics for diagnosis of invasive fungal disease. Clin Infect Dis 80:1095–1102. doi:10.1093/cid/ciaf021 39823293

[21] Donnelly JP, Chen SC, Kauffman CA, Steinbach WJ, Baddley JW, Verweij PE, Clancy CJ, Wingard JR, Lockhart SR, Groll AH, et al.. 2020. Revision and update of the consensus definitions of invasive fungal disease from the European organization for research and treatment of cancer and the mycoses study group education and research consortium. Clin Infect Dis 71:1367–1376. doi:10.1093/cid/ciz1008 31802125 PMC7486838

[22] Murugesan K, Hogan CA, Palmer Z, Reeve B, Theron G, Andama A, Somoskovi A, Steadman A, Madan D, Andrews J, Croda J, Sahoo MK, Cattamanchi A, Pinsky BA, Banaei N. 2019. Investigation of preanalytical variables impacting pathogen cell-free DNA in blood and urine. J Clin Microbiol 57:e00782-19. doi:10.1128/JCM.00782-19 31511335 PMC6813001

[23] Gouzien L, Che D, Cassaing S, Lortholary O, Letscher-Bru V, Paccoud O, Obadia T, Morio F, Moniot M, Cateau E, Bougnoux ME, Chouaki T, Hasseine L, Desoubeaux G, Gautier C, Mahinc-Martin C, Huguenin A, Bonhomme J, Sitbon K, Durand J, Alanio A, Millon L, Garcia-Hermoso D, Lanternier F, French Mycoses Study Group. 2024. Epidemiology and prognostic factors of mucormycosis in France (2012-2022): a cross-sectional study nested in a prospective surveillance programme. Lancet Reg Health Eur 45:101010. doi:10.1016/j.lanepe.2024.101010 39220434 PMC11363841

[24] Greenberg RN, Scott LJ, Vaughn HH, Ribes JA. 2004. Zygomycosis (mucormycosis): emerging clinical importance and new treatments. Curr Opin Infect Dis 17:517–525. doi:10.1097/00001432-200412000-00003 15640705

[25] Legrand M, Gits-Muselli M, Boutin L, Garcia-Hermoso D, Maurel V, Soussi S, Benyamina M, Ferry A, Chaussard M, Hamane S, Denis B, Touratier S, Guigue N, Fréalle E, Jeanne M, Shaal J-V, Soler C, Mimoun M, Chaouat M, Lafaurie M, Mebazaa A, Bretagne S, Alanio A. 2016. Detection of circulating mucorales DNA in critically ill burn patients: preliminary report of a screening strategy for early diagnosis and treatment. Clin Infect Dis 63:1312–1317. doi:10.1093/cid/ciw563 27535951

[26] Kontoyiannis DP, Wessel VC, Bodey GP, Rolston KV. 2000. Zygomycosis in the 1990s in a tertiary-care cancer center. Clin Infect Dis 30:851–856. doi:10.1086/313803 10852735

[27] Springer J, Lackner M, Ensinger C, Risslegger B, Morton CO, Nachbaur D, Lass-Flörl C, Einsele H, Heinz WJ, Loeffler J. 2016. Clinical evaluation of a Mucorales-specific real-time PCR assay in tissue and serum samples. J Med Microbiol 65:1414–1421. doi:10.1099/jmm.0.000375 27902424

[28] Bigot J, Godmer A, Prudenté L, Angebault C, Brissot E, Bige N, Voiriot G, Leger P-L, Petit-Hoang C, Atallah S, Gouache E, Senghor Y, Valot S, Hennequin C, Guitard J. 2022. Diagnosis of mucormycosis using an intercalating dye-based quantitative PCR. Med Mycol 60:myac015. doi:10.1093/mmy/myac015 35188208

[29] Imbert S, Portejoie L, Pfister E, Tauzin B, Revers M, Uthurriague J, Hernandez-Grande M, Lafon M-E, Jubert C, Issa N, Dumas P-Y, Delhaes L. 2023. A multiplex PCR and DNA-sequencing workflow on serum for the diagnosis and species identification for invasive aspergillosis and mucormycosis. J Clin Microbiol 61:e0140922. doi:10.1128/jcm.01409-22 36533925 PMC9879116

[30] Cruciani M, White PL, Mengoli C, Löffler J, Morton CO, Klingspor L, Buchheidt D, Maertens J, Heinz WJ, Rogers TR, Weinbergerova B, Warris A, Lockhart DEA, Jones B, Cordonnier C, Donnelly JP, Barnes RA, Fungal PCR Initiative. 2021. The impact of anti-mould prophylaxis on Aspergillus PCR blood testing for the diagnosis of invasive aspergillosis. J Antimicrob Chemother 76:635–638. doi:10.1093/jac/dkaa498 33374010

[31] Springer J, Lackner M, Nachbaur D, Girschikofsky M, Risslegger B, Mutschlechner W, Fritz J, Heinz WJ, Einsele H, Ullmann AJ, Löffler J, Lass-Flörl C. 2016. Prospective multicentre PCR-based Aspergillus DNA screening in high-risk patients with and without primary antifungal mould prophylaxis. Clin Microbiol Infect 22:80–86. doi:10.1016/j.cmi.2015.09.009 26400571

[32] Egger M, Jenks JD, Hoenigl M, Prattes J. 2020. Blood Aspergillus PCR: the good, the bad, and the ugly. J Fungi (Basel) 6:18. doi:10.3390/jof6010018 32012787 PMC7151127

[33] Skiada A, Pagano L, Groll A, Zimmerli S, Dupont B, Lagrou K, Lass-Florl C, Bouza E, Klimko N, Gaustad P, Richardson M, Hamal P, Akova M, Meis JF, Rodriguez-Tudela J-L, Roilides E, Mitrousia-Ziouva A, Petrikkos G, European Confederation of Medical Mycology Working Group on Zygomycosis. 2011. Zygomycosis in Europe: analysis of 230 cases accrued by the registry of the European Confederation of Medical Mycology (ECMM) Working Group on Zygomycosis between 2005 and 2007. Clin Microbiol Infect 17:1859–1867. doi:10.1111/j.1469-0691.2010.03456.x 21199154

[34] Park BJ, Pappas PG, Wannemuehler KA, Alexander BD, Anaissie EJ, Andes DR, Baddley JW, Brown JM, Brumble LM, Freifeld AG, Hadley S, Herwaldt L, Ito JI, Kauffman CA, Lyon GM, Marr KA, Morrison VA, Papanicolaou G, Patterson TF, Perl TM, Schuster MG, Walker R, Wingard JR, Walsh TJ, Kontoyiannis DP. 2011. Invasive non-Aspergillus mold infections in transplant recipients, United States, 2001-2006. Emerg Infect Dis 17:1855–1864. doi:10.3201/eid1710.110087 22000355 PMC3311117

