# Supplementary material for: Accuracy of plasma cell-free DNA PCR for non-invasive diagnosis of mucormycosis
Source: J Clin Microbiol. 2025 Jul 9;63(8):e00796-25. doi: 10.1128/jcm.00796-25 (PMC12345248; doi:10.1128/jcm.00796-25)
Supplement: Table S1 — Table shows clinical and laboratory data for patients with false-positive Mucorales plasma cfDNA PCR results. [file jcm.00796-25-s0001.docx]

**Supplementary Table 1.** Summary of clinical and microbiological data for patients with false-positive Mucorales plasma cfDNA PCR results.

| Patient Number | Underlying comorbidity | Infection site, EORTC Classification | Radiologic features | Histopathology results | Microbiology results | Treatment | Mortality 30 days / 6 months | Final Diagnosis |
| --- | --- | --- | --- | --- | --- | --- | --- | --- |
| 8 | Diabetes | Pulmonary,  no IFD | Diffuse consolidation and GGOs | Not performed | Positive Mucorales cfDNA PCR with CT 43.3, invasive sample testing not done | Not treated | Y / Y | Shock of unclear etiology |
| 9 | None | Pulmonary,  no IFD | Loculated pleural effusion | Granulomatous inflammation with positive Fite stain | Positive Mucorales cfDNA PCR with CT 41.8, invasive sample testing not done, negative pleural tissue culture | TB treatment | N / N | Tuberculosis |
| 10 | Lung transplant with BOS, metastatic head and neck SCC | Neck abscess,  no IFD | Neck abscess | Not performed | Positive Mucorales cfDNA PCR with CT 40.5, positive wound cultures for *Pseudomonas aeruginosa* | Remained on prophylaxis Itraconazole | N / Y | *Pseudomonas aeruginosa* neck abscess |
| 11 | DLBCL, neutropenia | Pulmonary,  no IFD | Bilateral GGOs and consolidative opacities | Negative for fungal elements, marantic endocarditis | Positive Mucorales cfDNA PCR with CT 40.5, Negative BAL culture, negative BAL mold PCR and GM, negative serum GM and B-D-Glucan. | L-Amphotericin | Y / Y | *Pneumocystis jirovecii* pneumonia based on PJP PCR |
| 12 | Decompensated cirrhosis | Liver,  no IFD | Normal lung parenchyma | Chronic cholecystitis | Positive Mucorales cfDNA PCR with CT 43.5, negative intestinal tissue culture, negative serum GM and B-D-Glucan | Not treated | N / N | Cirrhosis |
| 13 | ALL, allo-HSCT | Skin,  no IFD | Normal chest and sinus imaging | Negative | Positive Mucorales cfDNA PCR with CT 42.9, negative chest tissue culture, negative serum GM and B-D-Glucan | Isavuconazole | N / N | Suspected ponatinib-induced panniculitis |
| 14 | AML, neutropenia | Pulmonary,  no IFD | Peri-bronchovascular consolidations and GGOs | Not performed | Positive Mucorales cfDNA PCR with CT 42.2, negative serum GM and B-D-Glucan | Remained on prophylaxis Posaconazole | Y / Y | Recurrent aspiration |
| 15 | Metastatic colorectal cancer in remission | Pulmonary,  no IFD | Multifocal GGO and lobular consolidative opacities | Negative | Positive Mucorales cfDNA PCR with CT 36.9,  negative BAL Mold PCR and culture | Not treated | N / N | Aspiration pneumonia |
| 16 | ALL | Pulmonary,  no IFD | Multifocal patchy consolidations scattered tree-in-bud opacities | Not performed | Positive Mucorales cfDNA PCR with CT 36.5, 2 negative plasma Mucorales cfDNA PCR | L-Amphotericin | N / N | Multifocal pneumonia |
| 17 | AML, neutropenia | Unknown,  no IFD | No evidence of infection | Not performed | Positive Mucorales cfDNA PCR with CT 38.1, negative serum GM and B-D-Glucan | Remained on prophylaxis posaconazole | N / N | *Streptococcus mitis* bacteremia |
| 18 | Lung cancer, multiple myeloma, auto-HSCT, neutropenia | Pulmonary,  no IFD | Diffuse bilateral GGO and pleural effusion | Atypical carcinoid lung tumor | Positive Mucorales cfDNA PCR with CT 40.0, negative pleural fluid Mold PCR and culture, negative serum GM and B-D-Glucan | Not treated | Y / Y | Pulmonary edema and metastatic cancer |
| 19 | Mycoides Fungoides, allo-HSCT | Pulmonary,  no IFD | Diffuse GGOs and consolidative opacities | Not performed | Positive Mucorales cfDNA PCR with CT 38.4, positive B-D-Glucan (90 pg/mL), negative BAL Mold PCR and culture, and negative serum GM | Remained on prophylaxis posaconazole | N / N | MRSA and *Corynebacterium striatum* HAP |

BOS – bronchiolitis obliterans syndrome; SCC – squamous cell carcinoma; DLBCL – diffuse large B-cell lymphoma; ALL – acute lymphocytic leukemia; HSCT – hematopoietic stem cell transplant; AML – acute myelogenous leukemia; GGO – ground glass opacities; HAP – hospital acquired pneumonia; GM – galactomannan; IFD – invasive fungal disease; MRSA – methicillin-resistant *Staphylococcus aureus*; CT – cycle threshold
